# CD8^+^ T-cell immunity orchestrated by iNKT cells

**DOI:** 10.3389/fimmu.2022.1109347

**Published:** 2023-01-18

**Authors:** Yingyu Qin, Xueyang Bao, Mingzhu Zheng

**Affiliations:** Department of Pathogenic Biology and Immunology, Jiangsu Provincial Key Laboratory of Critical Care Medicine, School of Medicine, Southeast University, Nanjing, Jiangsu, China

**Keywords:** invariant NKT (iNKT) cell, CD8^+^ T cell, cross-priming, memory, aging

## Abstract

CD8^+^ T cells belonging to the adaptive immune system play key roles in defending against viral infections and cancers. The current CD8^+^ T cell-based immunotherapy has emerged as a superior therapeutic avenue for the eradication of tumor cells and long-term prevention of their recurrence in hematologic malignancies. It is believed that an effective adaptive immune response critically relies on the help of the innate compartment. Invariant natural killer T (iNKT) cells are innate-like T lymphocytes that have been considered some of the first cells to respond to infections and can secrete a large amount of diverse cytokines and chemokines to widely modulate the innate and adaptive immune responders. Like CD8^+^ T cells, iNKT cells also play an important role in defense against intracellular pathogenic infections and cancers. In this review, we will discuss the CD8^+^ T-cell immunity contributed by iNKT cells, including iNKT cell-mediated cross-priming and memory formation, and discuss recent advances in our understanding of the mechanisms underlying memory CD8^+^ T-cell differentiation, as well as aging-induced impairment of T-cell immunity.

## Introduction

CD8^+^ T lymphocytes function as a key component of the adaptive immune system and are critical for combating intracellular pathogens and cancer cells (1, 2). Following acute infection, CD8^+^ T cells can be typically divided into three phases (3). During the first phase (priming phase), CD8^+^ T cells differentiated into heterogenic effector cells with proliferation, cytokine production, and cytotoxic functions (4, 5). Regarding the potential of memory cell differentiation, the effector compartments are roughly divided into memory precursor effector cells (MPECs) and short-lived effector cells (SLECs), which can be distinguished by the surface markers KLRG1 and CD127 (6, 7). With the pathogens cleared, the second phase (contraction phase) ensues, and most effector cells during robust contraction undergo activation-induced cell death. Only 5%–10% of CD8^+^ T cells survive and enter the memory phase, with long-term maintenance and self-renewal abilities (8, 9).

Natural killer T (NKT) cells are an innate-like T-cell subset that shares both physical and functional characteristics with NK cells and T cells (10). Unlike conventional αβT lymphocytes that recognize protein peptides presented by MHC class I or class II, NKT cells utilize their T-cell receptors (TCRs) to recognize self or foreign lipid antigens presented by a non-classical MHC class I-like molecule, CD1d (11, 12). According to their TCR repertoire, NKT cells are classified into type I and type II NKT cells. Type I NKT cells, also termed invariant NKT (iNKT) cells, express invariant TCR α chain (Vα14-Jα18 chain in mice; Vα24-Jα18 chain in humans) pairing with a limited spectrum of β chains (Vβ8.2, Vβ2, and Vβ7 in mice; Vβ11 in humans) (10, 13). iNKT cells are the most broadly studied subset of NKT cells that can be activated by α-galactosyl ceramide (α-Galcer), a marine sponge *Agelas mauritiana*-derived lipid (14), whereas type II NKT cells are α-Galcer non-reactive cells displaying more heterogeneous αβ chains of TCR. So far, the knowledge of type II NKT cells is less identified because they lack specific markers. The well-known role of Type II NKT cells is their suppressive immune response, especially in tumor immunity (15). In this review, we will focus on iNKT cells.

The population of iNKT cells is much smaller than that of conventional T cells. In mice, iNKT cells are 0.5% of lymphocytes in peripheral blood, 1%–2% in the spleen, and 20%–30% in the liver (16, 17). However, the number of iNKT cells in humans is significantly less and is discrepant among individuals. In humans, NKT cells are only 0.05%–1% of lymphocytes in the liver and 0.01%–0.1% of lymphocytes in peripheral blood generally, while it is up to 3% of peripheral blood mononuclear cells in some individuals (18–22). Even though iNKT cells are such a small sub-population compared with conventional peptide reactive T cells, several unique properties are positioned as a crucial regulatory population to influence diverse immune responses. First, their TCR repertoire is very limited, which contributes to the number of iNKT cell precursors being reactive to a certain lipid antigen being much higher than that of certain peptide-reactive T-cell precursors. Second, iNKT cells are autoreactive by responding to self-lipids (23). iNKT cells develop and differentiate into distinct subsets that are analogous to T helper 1 (Th1), Th2, and Th17 subsets in the thymus and move to peripheral lymphoid and non-lymphoid organs where they are further activated (24). These unique properties stimulate iNKT cells to respond to infection and inflammation through TCR engagement and/or cytokine signals within hours, with the production of a large amount of diverse cytokines to regulate innate and adaptive immune responders, such as neutrophils, NK cells, dendritic cells, macrophages, B cells, and T cells (25–30).

A large body of work has attempted to elucidate the regulatory roles of iNKT cells in affecting the response of CD8^+^ T cells including their effector functions and fate decisions. Among them, CD4^+^ T cell-mediated help is largely studied (31, 32). Like CD4^+^ T cells, iNKT cells also play an important role in the impact of CD8^+^ T-cell immunity. In this review, we will summarize current knowledge about the roles of iNKT cells in regulating CD8^+^ T-cell response, including cross-priming, effector function, and memory differentiation.

## iNKT cell activation and subsets of iNKT cells

**Figure 1:**
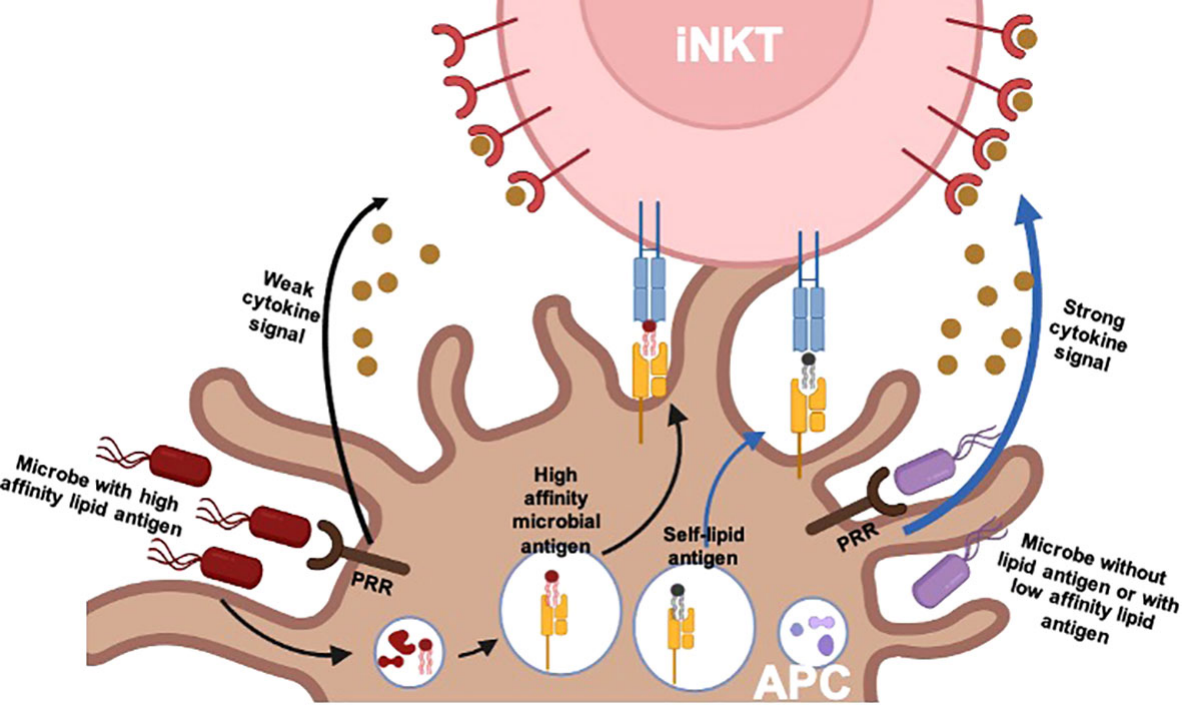
TCR and/or cytokine-driven iNKT cell activation. There are two pathways involved in the physiological activation of iNKT cells: 1) for pathogens expressing affinity lipid antigens, iNKT cell activation is dominantly driven by TCR signals provided by the complex of high-affinity microbial lipid antigen presented by CD1d of an APC. It is less dependent on additional cytokine signals, which are provided by APCs after stimulation of pattern recognition receptors (PRRs). 2) For pathogens lacking lipid antigens or with low-affinity lipid antigens, iNKT cell activation is dominantly driven by cytokine signals. The engagement of microbial products with PRRs leads to APC production of pro-inflammatory cytokines such as IL-12. In this case, TCR signals are commonly still required, which is provided by self-lipid or low-affinity lipid antigens. TCR, T-cell receptor; iNKT, invariant natural killer T; APC, antigen-presenting cell.

There are different ways to activate iNKT cells. Like conventional T cells, iNKT cells can be activated *via* the engagement of TCR with a glycolipid–CD1d complex. The first lipid antigen, α-Galcer, was discovered by Kawano and colleagues 25 years ago, which can potently activate both mouse and human iNKT cells (14). Thereafter, most knowledge on the function of iNKT cells came from it or its synthetic analog KRN7000. Upon activation, iNKT cells can rapidly produce a large amount of cytokines, such as IFN-γ, TNF-α, interleukin (IL)-2, IL-4, IL-5, IL-3, IL-13, IL-10, IL-9, IL-17, IL-21, and IL-22 (33, 34). Stimulated iNKT cells can also secrete diverse chemokines, including monocyte chemoattractant protein (MCP)-1, RANTES (regulated on activation, normal T cell expressed and secreted), macrophage inflammatory protein (MIP)-1α and MIP-1β, and eotaxins (35–38). Additionally, microbial lipid antigens also have been extensively identified, such as α-glucosyl diacylglycerols (αGlc-DAGs) from *Streptococcus pneumoniae* and group B *Streptococcus*, α-glucuronosylceramides and α-galacturonosylceramides from *Sphingomonas* spp., α-galactosyldiacylglycerols (α-GalDAGs) from *Borrelia burgdorferi*, and phosphatidylinositol mannosides (PIMs) from *Mycobacterium tuberculosis* (39–43). These microbe-derived glycolipids sustained iNKT cell activation through engagement of their invariant TCRs (**Figure 1**). The high-affinity lipids such as α-glucuronosylceramides elicit iNKT cell production of IFN-γ and IL-4 similar to α-Galcer (39). In the absence of pathogen-associated lipid antigens, or lipid antigens with low affinity, cytokine-driven activation of iNKT cells is dominant (44, 45). iNKT cells can be activated by antigen-presenting cells (APCs) that have been stimulated by Toll-like receptor agonists in the absence of infection, and this activation requires a lipid–CD1d complex, which suggests that self-lipids can contribute to iNKT cell activation (44, 46, 47). This activation requires iNKT cells to receive the signals from the complex of TCR–self-lipid–CD1d and pro-inflammatory cytokines, such as IL-12, IL-18, IL-23, and IL-25, released from APCs. Even in the absence of TCR engagement, iNKT cells can also be activated by responding to pro-inflammatory cytokines due to their high expression of the cytokine receptors in the steady state (44). IL-12 is the best-described cytokine mediator of iNKT cell activation. In some bacterial and viral infections, the stimulated APCs produce a large amount of IL-12, which is sufficient to activate iNKT cells, whereas without IL-12 production, APCs cannot activate iNKT cells, even in the presence of identified microorganism-derived lipid antigens, which indicates that cytokine signaling plays a crucial role in iNKT cell activation (48–50). In addition, cytokine-induced activation results in iNKT cell production of IFN-γ but not IL-4 (44, 51), which suggests that IL-12 may induce the activation of a subset of iNKT cells, or it only provides a weak stimulatory signal due to failure of proliferation. Furthermore, it is also found that IL-18 signaling is essential for splenic iNKT cells rather than liver iNKT cells during cytomegalovirus (CMV) infection (50), which implies that cytokine signaling may affect not only iNKT cell response but also localization.

To date, mouse iNKT cells have been classified into five effector subsets on the basis of their specific markers referring to cytokine production and transcriptional factor signatures. Analogous to Th cell subsets, they include NKT1, NKT2, NKT17, iNKT10, and follicular help NKT (iNKT_FH_). NKT1 cells are enriched in the spleen and liver and express Th1 cell-associated transcription factor T-bet. However, unlike Th1 cells, they express both type 1 and type 2 cytokines (52, 53). NKT2 cells are primarily located in the medullary area of the thymus and T-cell zone of the spleen and mesenteric lymph nodes as well as the lungs. NKT2 cells can produce IL-4 and IL-13 and therefore resemble Th2 cells (53). Similar to Th17 cells, IL-17A-producing iNKT cells are termed NKT17, which are primarily located in the lymph nodes, skin, and lungs (53). NKT_FH_ cells are similar to T_FH_ cells that are primarily located in the spleen and act in germinal centers to promote affinity maturation of antibodies. iNKT10 cells are IL-10-producing cells with a regulatory phenotype but without foxp3 expression (54). Therefore, the regulation role of iNKT cells in the modulation of other immune cells may be partly associated with diverse cytokine production by functionally distinct subsets of these cells, and the different tissue-homing preferences of these iNKT cell subsets may also ultimately influence local immune responses. Mitchell Kronenberg and colleagues have recently provided an excellent in-depth review of these subsets of iNKT cells (24).

## Cross-priming of CD8^+^ T cells mediated by iNKT cells

Priming of naïve T cells requires three key signals: TCR engagements, costimulatory signals provided by CD28 and TNF receptor family, and cytokine signals. Dendritic cells (DCs) are APCs responsible for providing the three signals of cross-priming of CD8^+^ T cells. Cross-priming is a process that permits specialized DCs to cross-presentation of extracellular antigens to CD8^+^ T cells conferring their effector functions to defend against tumors and intracellular pathogenic infections. This immunogenic cross-presentation requires the presence of pathogen-derived molecule patterns and/or CD4^+^ T help cells, a process termed as “licensing” of DCs, which prevents unwanted immune responses for the innocuous antigens or autoantigens (31, 55). Classically, the licensing is mediated through CD40–CD40L interactions of CD4^+^ T help cells with DCs, which is the primary mechanism that supports CD8^+^ T-cell response by CD4^+^ T cells (56, 57). However, a growing number of studies have suggested that iNKT cells are another subset for DC licensing (30, 58). Consistent with CD4^+^ T-cell licensing, iNKT cell-mediated DC licensing also allows DC functional maturation in a CD40L-dependent manner (59). After iNKT cells recognize their cognate self or foreign lipid antigens displayed by CD1d of APCs, immediate activation of iNKT cells induces transient upregulation of CD40L on iNKT cells and subsequently activates DCs *via* interaction of CD40L with CD40. The cross-talk between iNKT cells and DCs *via* CD40–CD40L interaction triggers DC functional maturation resulting in the upregulation of costimulatory proteins and IL-12 production, which further activate iNKT cells (60, 61). Additionally, TNF-α and IFN-γ released by DCs and iNKT cells, respectively, assist in CD40–CD40L-mediated DC licensing. As a consequence, CD8^+^ T-cell response is promoted by the licensed DCs through interaction with the costimulatory proteins and inflammatory signals (61, 62). CD27 is a member of TNFR family, with stimulation of CD27 signals *via* interaction with CD70. Distinct from CD28, CD27 costimulation signal not only promotes CD8^+^ T-cell effector differentiation but also enhances their survival (63, 64). This survival relies completely on IL-2R signaling and autocrine IL-2 production (63). Additionally, CD4^+^ T cell-mediated help *via* CD27 costimulation is also involved in memory CD8^+^ T-cell development (65, 66), as well as in preventing CD8^+^ T cells from tolerance by reducing PD-1 expression (67). Consistent with CD4^+^ T cell-mediated help, the interaction of CD70 and CD27 is also crucial for iNKT cell-mediated promotion of CD8^+^ T-cell response including CD8^+^ T cell-mediated antitumor immunity (62).

When iNKT cells are primed, they will accumulate in the marginal zone of the spleen, where they co-interact with CD8α^+^ conventional DCs (CD8α^+^cDCs) with a unique expression of chemokine receptor XCR1, a major type of APCs responsible for cross-priming (68, 69). After initial activation, CD8^+^ T cells will recruit other types of cells to the site of initial antigen recognition to create their own optimal priming microenvironment. Plasmacytoid DCs (pDCs) are another subset of DCs playing a critical role in anti-viral infections *via* the production of type I interferon (IFN) (70). Shin-ichiro Fujii and colleagues suggested that iNKT cell-mediated DC (XCR1^+^DC) licensing for cross-priming of CD8^+^ T cells is through induction of the cross-talk between pDCs and XCR1^+^ DCs (71). This cross-talk is important for the cross-presentation of XCR1^+^ DCs, which is further supported by later studies (72–74). Cell-mediated licensing requires the same DC to physically interact with the help cells and CD8^+^ T cells. Such interactions are usually governed by chemokines and their receptors. For CD4^+^ T cell-mediated help, the DCs licensed by CD4^+^ T cells express a high level of CCR5 ligands and recruit CCR5-expressing CD8^+^ T cells (naïve and effector) and process cross-priming. Unlike CD4^+^ T cell-mediated help, iNKT cell-licensed DCs produce CCL17 to attract CCR4 (CCL17 receptor) expressing naïve CD8^+^ T cells (75, 76). Nine years later, they further extend this notion that iNKT cell-mediated induction of CXCR3 and CCR4 expression on CD8^+^ T cells could affect the fate of CD8^+^ T cells (77). Consistently, a recent study shows that iNKT cells can promote the generation of functional CXCR3^+^CCR4^+^CD8^+^ T cells, which mediate rapid rejection of allogeneic hepatocytes engrafted in the liver (78). Moreover, on DCs, CD1d molecules are expressed in all hematopoietic cells, including monocytes, B cells, and T cells (11, 79). Our study showed that there is a direct interaction between TCRs (iNKT cells) and CD1d molecules (CD8^+^ T cells), which promotes CD8^+^ T-cell activation in a DC-independent manner (80).

## The formation of memory CD8^+^ T cell assisted by iNKT cells

Immunological memory is established after successful priming, which is a fundamental feature of the acquired immune response. Clearly, the generation of long-lived memory CD8^+^ T cells is one of the primary goals in the development of therapeutic vaccines. The role of iNKT cells in the promotion of CD8^+^ T-cell response as well as memory formation in immunization, pathogenic infection, and tumor immunity in mice or humans has largely been addressed (81, 82). Considering that α-Galcer provokes strong iNKT cell activity in both mice and humans, it has long been used as a prototypical immune adjuvant co-administrated with antigens of interest in the arrangement of experimental and clinical settings. Some of the most extensive studies are using inactivated influenza virus as the source of antigen with co-administration of α-Galcer for iNKT cell activation, which significantly enhances both cellular and humoral responses (83). Injection of soluble α-Galcer limited the immediate CD8^+^ T-cell response but promoted the survival of long-lived memory populations, which are capable of protection from the influenza A virus challenge (84). In another study, it was suggested that α-Galcer conjugated with the peptides of influenza A virus-associated protein induces not only robust effector CD8^+^ T-cell response but also memory CD8^+^ T-cell generation (85). The difference in CD8^+^ T-cell priming/effector function may be attributed to the α-Galcer and antigen peptides accessing the same APCs to enable licensing events to occur. A similar protective effect was also shown in a mouse cytomegalovirus (MCMV) infection model. Injection of soluble α-Galcer increased central memory CD8^+^ T-cell differentiation (86). In tumor models, studies have shown that α-Galcer-loaded tumor cells induce a strong antitumor immunity along with memory cytotoxic T-lymphocyte (CTL) generation in a DC-dependent manner (87) as well as a recent popular therapeutic avenue, CAR-iNKT cells, which present superior antitumor effects along with the generation of antitumor central memory CD8^+^ T cells (81). Additionally, some studies also show that activation of autologous iNKT cells induces strong antitumor effects of CD8^+^ T cells including prevention of CD8^+^ T-cell exhaustion (88–91). Resident memory T cells are crucial for local immunity and recall response, which is considered more potential subsets in vaccine designation (92). A recent study shows that induction of iNKT cell activation can promote the generation of liver-resident memory CD8^+^ T cells, which prevents malaria (93). In naïve mice raised in specific pathogen-free and germ-free conditions, a small CD8^+^ T-cell population displaying memorial phenotypes is named “virtual memory” CD8^+^ T cells (CD8^+^ T_VM_). Some studies have shown that IL-4-producing iNKT cells increase the abundance of CD8^+^ T_VM_ (94–96). The increased CD8^+^ T_VM_ cells could provide broad protection (94). In conclusion, activation of iNKT cells not only can promote CD8^+^ T-cell response but also has a high potential in helping memory T-cell generation.

**Figure 2:**
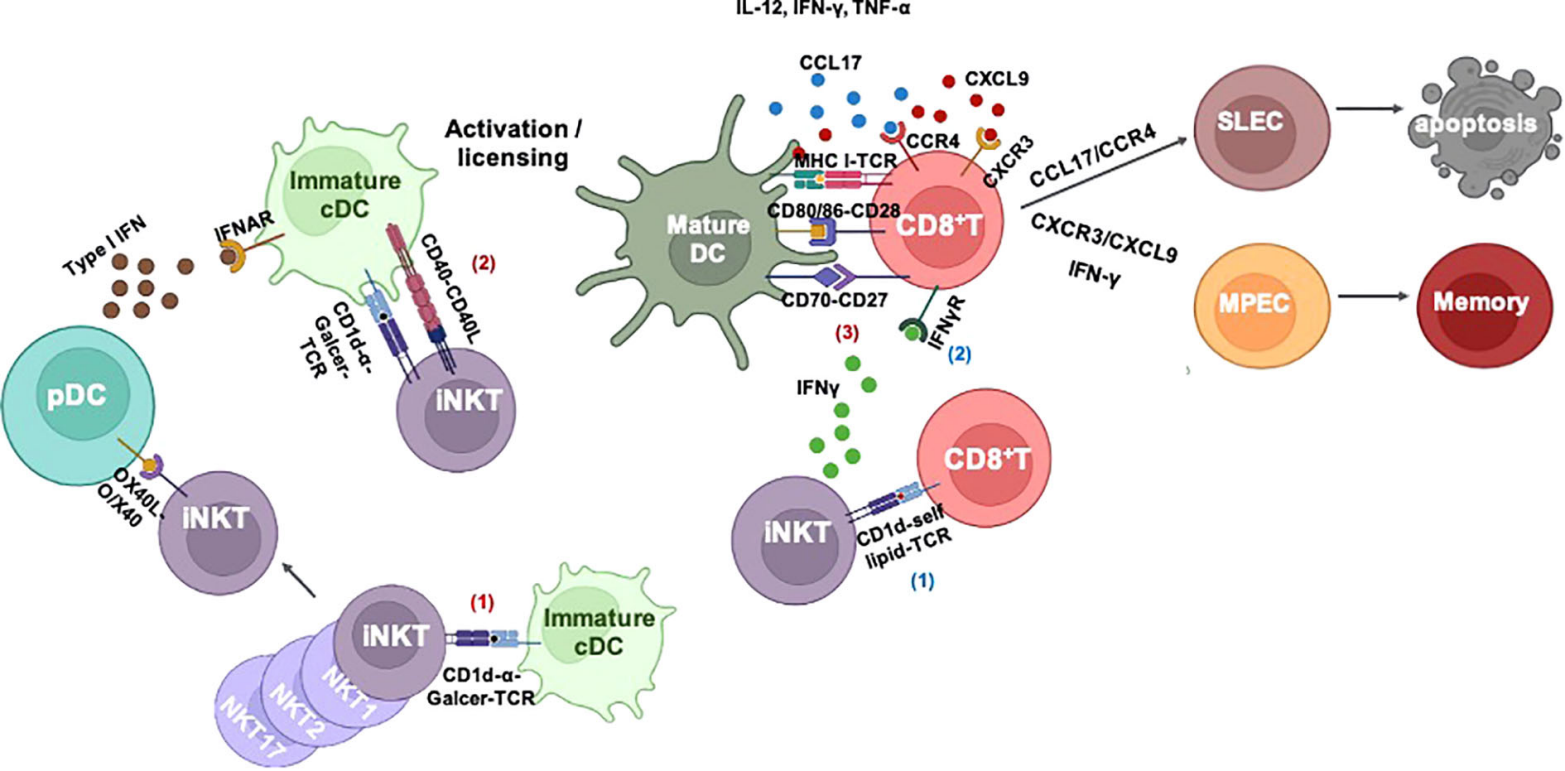
iNKT cell-mediated CD8^+^ T-cell immunity. I) iNKT cell-mediated cross-priming of CD8^+^ T cells after responding to α-Galcer: 1) iNKT cells are activated by α-Galcer without any costimulatory signals. 2) Immature DCs capture antigens and interact with activated iNKT cells to undergo iNKT cell-induced maturation *via* CD40–CD40L and inflammatory cytokine IFN-γ and TNF-α. Activated iNKT cells can also activate plasmacytoid DC (pDC) *via* OX40L–OX40 signaling; thereafter, pDC assists DC maturation and cross-priming of CD8^+^ T cells in a type I IFN-dependent manner. iNKT cell-mediated DC licensing promotes DC cross-presentation of antigens to CD8^+^ T cells *via* upregulation of costimulatory signals such as CD80/86–CD28, CD70–CD27, and cytokines IL-12, IFN-γ, and TNF-α. II) Without exogenous lipid antigen: iNKT cells can be activated by CD8^+^ T cells through CD1d–self lipid–TCR engagement. Activated iNKT cells enhance CD8^+^ T-cell effector function in an IFN-γ dependent manner. III) iNKT cell-mediated CD8^+^ T-cell fate decision: 1) iNKT cell-mediated cross-talk of pDC–cDC–CD8^+^ T cells promotes memory CD8^+^ T-cell development. 2) iNKT-mediated cDC licensing SLEC development *via* CCL17/CCR4 or MPEC development *via* CXCR3/CXCL9 and IFN-γ. iNKT, invariant natural killer T; DCs, dendritic cells; TCR, T-cell receptor; SLEC, short-lived effector cell; MPEC, memory precursor effector cell.

The knowledge of mechanisms of iNKT cells in help for memory CD8^+^ T-cell differentiation is critical for the development of new vaccination strategies. Unlike a large body of investigations in CD4^+^ T cell help for memory CD8^+^ T-cell formation, the studies about iNKT cell-mediated help are still limited. There are several studies that have tried to explore the potential mechanisms of iNKT cells in affecting memory CD8^+^ T-cell memory differentiation, which is concluded in **Figure 2**. pDCs are well known as an additional subset of DCs supporting conventional DC cross-priming of CD8^+^ T cells in a type I IFN-dependent manner (72, 74). An early study shows that the cross-talk between pDC and cDC, which is mediated by activated iNKT cells, is important for memory CD8^+^ T-cell formation (71). A recent study provides evidence that there is a cross-talk among iNKT cell, DC, and CD8^+^ T cells, which is involved in CD8^+^ T-cell memory programming (77). In line with our findings, the live imaging showed the presence of direct interaction of iNKT cells and CD8^+^ T cells at the early stage (80). A consistent cross-talk among iNKT cell, CD8^+^ T cell, and DC is formed at a later stage, and the fate of CD8^+^ T-cell memory differentiation is regulated by CXCR3/IFN-γ and IL-4/CCL17/CCR4 axes, which drive CD8^+^ T-cell differentiation into MPECs and SLECs, respectively. However, in contrast to this notion, the CXCR3 has been believed to drive CTL toward effector fate rather than memory fate (97, 98), whereas CCR4 is associated with memory T help cell development and phenotypes of memory CD8^+^ T cells (99–101). In addition to the above studies, an early study suggests that activation of iNKT cells enhances memory CD8^+^ T-cell homeostatic proliferation depending on IL-4-STAT6 signaling (95); similar to this study, IL-4-producing Kruppel-like factor (KLF) 13-positive iNKT cells is critical for thymic memory like CD8^+^ T-cell formation (96).

In conclusion, since iNKT cells rapidly produce diverse cytokines and chemokines and also can interact with APCs and T cells directly, the roles of iNKT cells in CD8^+^ T-cell fate regulation are intricate, which still remains largely unknown, as well as the mechanism.

## Age-associated decline of T-cell immunity

A significant characteristic of the aging process is age-associated immune dysfunction, which results in an increase in susceptibility to infection, cancer, and autoimmune diseases. Reduction of the TCR repertoire, imbalance of naïve and memory compartments, T-cell senescence, and reduced proliferation and cytokine production are considered to be the hallmarks of T-cell aging (102, 103). The most notable changes are in CD8 rather than in CD4 T cells since the latter maintain their populations through homeostatic proliferation (104). Clearly, aging-induced alteration in either numbers or functions of iNKT cells could affect the quality of T-cell response. Several studies have reported that the number/frequency of NKT cells is increased in older mice and human beings (105–107). This increase exhibits an unusual effect of aging, as other types of immune cells decrease or are constant in number. Additionally, it is also found that the increased NKT cells originate from newly made cells. In contrast, it has been reported that Vα14 and Vα24 NKT cells are decreased in aged mice and humans, particularly in the liver (108, 109). A recent study also shows that aging changes the subset composition of iNKT cells, as well as decreases their proliferative capacity (110). Collectively, the mechanisms responsible for the increase or decrease of NKT cells in aged mice remain to be elucidated. In addition to changes in the number of NKT cells, the cytokine production of NKT cells is also changed in aged mice. Several data support the notion that NKT cells tend to differentiate to an immunosuppressive phenotype by an increase in IL-4 and IL-10 production in older mice and humans (105, 107, 111). The effects of NKT cells to modulate several aspects of T-cell function are different in aged mice. The suppressive functions of NKT cells on the effector phase of T-cell immunity including proliferation and cytokine expression were observed in aged mice (105, 112). As previously described, virtual memory T cells (T_VM_) are antigen naïve T cells, which are generated by homeostatic proliferation. Studies have reported that IL-4 is involved in the generation of T_VM_, and the thymic NKT pool is critical for enlarging frequencies of IL-4-producing cells (94, 95, 113, 114). Over the course of aging, T_VM_ is accumulated (115). This age-related accumulation of T_VM_ decreases the primary response of CD8^+^ T cells due to the development of senescence in T_VM_ (116). Therefore, regardless that NKT cells affect T_VM_ generation, the senescence of T_VM_ in the context of aging should also be elucidated. Although relatively meager, the current set of studies supports the notion that aging affects the number and function of NKT cells, which will provoke a cascade of decreased innate and adaptive immune responses including CD8^+^ T-cell response, with the subsequent appearance of age-related diseases.

## The natural characteristics of iNKT cell help

A notion that should be considered is that the knowledge of iNKT cell-mediated help for CD8^+^ T-cell response or memory formation is largely concluded by α-Galcer, a potent synthetic glycosphingolipid antigen. It may not represent all functions of iNKT cells. Examples presented in models of dextran sulfate sodium (DSS)-induced colitis show that iNKT cells suppress pathogenic NK1.1^+^CD8^+^ T cells *via* expansion Treg cells using CD1d or Jα18 knockout mice (117). Foreign lipid antigens are not always present. Indeed, most microorganisms lack cognate lipid antigens, as well as cancer and autoimmune diseases (118). The critical roles of iNKT cells in these contexts have been largely addressed without using the exogenous α-Galcer treatment. As discussed above, iNKT cells are activated by self-lipids and/or pro-inflammatory cytokines. A small number of self-lipid antigens have been explored, which are generally divided into glycosphingolipids and phospholipids, including isoglobotrihexosylceramide (iGb3) (119), β-glucosylceramide (β-GlcCer) (120), α-glycosylceramides (121), lysophosphatidylcholine (lysoPC) (122), plasmalogen lysophosphatidylethanolamine (pLysoPE), and lysophosphatidic acid (eLPA) (123). Although these self-lipids have been identified, their potential functions including whether they regulate CD8^+^ T-cell response are still largely unknown. Recent studies showed that self-lipids presented endoplasmic reticulum (ER)-stressed APCs can potently activate iNKT cells as α-Galcer dose (124), while some studies suggested that self-lipid and/or cytokine-driven stimulation-induced iNKT cell production of IFN-γ but not IL-4 (44, 51, 80). For regulation of CD8^+^ T-cell response, Albert Bendelac and colleagues suggested that the response of iGb3 enhances the cross-priming of CD8^+^ T cell-like α-Galcer dose (75). Se-Ho Park and colleagues also supported the notion that self-lipid-activated iNKT cells strengthen the primary response and secondary response of CTLs, while the potential self-lipid is not explored (80, 125). The optimal roles of iNKT cells in strengthening the antitumor response of CTLs were also determined (80). Further study to explore the physiological role of iNKT cells in regulating CD8^+^ T-cell immunity in the context of viral infection and cancers is necessary for the improvement of immunotherapy in chronic viral infectious diseases and cancers.

## Concluding remarks

iNKT cells are a manipulated subset of T lymphocytes, with diverse regulatory functions that affect the effector function and fate of CD8^+^ T cells, which are attributed to their ability to release a rapid burst of cytokines and chemokines without the need for clonal expansion and differentiation, as well as providing indispensable costimulatory signals for licensing of DCs. Compared with CD4^+^ Th cells, iNKT cells play non-redundant roles in supporting CD8^+^ T-cell optimal priming and secondary response or regulating CD8^+^ T-cell fate decision (death or memory development) at their interaction timing, site, and subsets of iNKT cells. Additionally, significant changes in numbers and functions are determined in iNKT cells with age, and limited research has found that iNKT cells impair T-cell immunity. Further studies involving iNKT cell-mediated CD8^+^ T-cell immunity, especially in the modulation of antigen-experienced memory T-cell development, and antigen naïve T_VM_ formation in both young and aged conditions need to be widely explored. Meanwhile, the physiological role of iNKT cells responding to natural lipid and/or inflammatory cytokines in critical diseases, such as chronic viral infectious disease and cancers, in which both CD8^+^ T and iNKT cells are laying critical roles, still needs to be elucidated.

## Author contributions

YQ prepared the manuscript. XB critically reviewed the manuscript. MZ edited and finalized the manuscript. All authors contributed to the article and approved the submitted version.
